# Associations of the APOE ε2 and ε4 alleles and polygenic profiles comprising APOE-TOMM40-APOC1 variants with Alzheimer’s disease biomarkers

**DOI:** 10.18632/aging.204384

**Published:** 2022-11-17

**Authors:** Alexander M. Kulminski, Ethan Jain-Washburn, Elena Loiko, Yury Loika, Fan Feng, Irina Culminskaya, for the Alzheimer’s Disease Neuroimaging Initiative

**Affiliations:** 1 Biodemography of Aging Research Unit, Social Science Research Institute, Duke University, Durham, NC 27705, USA; 2 Center for Imaging of Neurodegenerative Disease, University of California, San Francisco, CA 94143, USA

**Keywords:** aging, apolipoprotein E polymorphism, Alzheimer’s disease, haplotypes, Alzheimer’s disease biomarkers

## Abstract

Capturing the genetic architecture of Alzheimer’s disease (AD) is challenging because of the complex interplay of genetic and non-genetic factors in its etiology. It has been suggested that AD biomarkers may improve the characterization of AD pathology and its genetic architecture. Most studies have focused on connections of individual genetic variants with AD biomarkers, whereas the role of combinations of genetic variants is substantially underexplored. We examined the associations of the APOE ε2 and ε4 alleles and polygenic profiles comprising the ε4-encoding rs429358, TOMM40 rs2075650, and APOC1 rs12721046 polymorphisms with cerebrospinal fluid (CSF) and plasma amyloid β (Aβ40 and Aβ42) and tau biomarkers. Our findings support associations of the ε4 alleles with both plasma and CSF Aβ42 and CSF tau, and the ε2 alleles with baseline, but not longitudinal, CSF Aβ42 measurements. We found that the ε4-bearing polygenic profiles conferring higher and lower AD risks are differentially associated with tau but not Aβ42. Modulation of the effect of the ε4 alleles by TOMM40 and APOC1 variants indicates the potential genetic mechanism of differential roles of Aβ and tau in AD pathogenesis.

## INTRODUCTION

Late-onset Alzheimer’s disease (AD) has a multifactorial etiology, which is affected by a complex interplay of genetic and non-genetic factors [1]. The estimates of heritability of 45% for women and 58% for men in a study of Swedish twins [2] suggest that the genetic contribution to AD pathogenesis can be substantial. However, capturing the genetic architecture of AD is challenging because of the complex interplay of genetic and non-genetic factors in its etiology. Indeed, despite discoveries of AD loci in large-scale genome-wide association studies (GWAS) [3–6], these loci are considered risk rather than causal factors for AD. The challenging role of genes in AD is exemplified by the apolipoprotein E (*APOE*) ε4 allele, which is the strongest individual genetic risk factor for AD. Even though the *APOE* gene has been studied for decades, its role in AD is not fully understood [7, 8].

The 2018 NIA-AA research framework [9–11] promoted the biological definition of AD pathology based on amyloid β (Aβ), tau, and neurodegeneration biomarkers. Positron emission tomography (PET) imaging studies identified Aβ and tau as valuable biomarkers to characterize AD development, with tau considered a more accurate AD biomarker than Aβ [12]. Cerebrospinal fluid (CSF) and plasma measurements of Aβ, particularly Aβ42 and tau, are used to facilitate AD diagnosis [13, 14]. Low levels of Aβ42 indicate accumulation of Aβ in plaques, whereas high tau levels are associated with neuronal injury [15]. The emergence of the biological definition of AD pathology opens a promising avenue in studies of the genetic architecture of AD.

Prior analyses showed that carriers of the *APOE* ε4 allele have lower levels of Aβ42 [16] and higher levels of tau [17] in CSF, although the latter might be controversial [18]. Given the multifactorial etiology of AD, the genetic architecture of AD and its biomarkers is likely heterogeneous. It is complicated by polygenicity, pleiotropy, and interactions with genetic and non-genetic factors. This complexity is in contrast to Mendelian traits; the traits, which may be caused by genetic mutations directly affecting protein function [19]. For example, the autosomal dominant form of early-onset AD can be caused by specific mutations in the *APP*, *PSEN1*, or *PSEN2* genes [20–23].

Studies also examined a role of an interplay between the *APOE* variants and other genetic factors in AD. For example, Franceschi’s group identified a haplotype comprising the ε4 and rs405509_T promoter variants conferring the AD risk [24]. Haplotypes composed of non-coding variants in the *APOE* gene cluster were reported in [25, 26]. Roses’s group showed a pivotal role of *TOMM40* poly-T rs10524523 and *APOE* ε2/ε3/ε4 haplotypes in AD pathogenesis [27]. Le Guen et al., [28] identified rare *APOE* functional variants co-inheriting with the ε4 allele and ameliorating its adverse effect. However, studies examining the relationships between combinations of genetic variants and AD biomarkers are in their infancy [29] and novel methods can be helpful for accelerating progress in the field.

A method examining differences in linkage disequilibrium (LD) structures in trait-affected and unaffected subjects was suggested to efficiently map promising associations [30]. Its advantage is that it helps identify connections between combinations of genetic variants and a complex trait. This method is well adapted to examine pairs of genetic variants, such as single nucleotide polymorphisms (SNPs). Recently, we generalized it from pairs of SNPs to triples of SNPs using the co-skewness metric [31]. Following these methods, we mapped compound genotypes—certain combinations of genetic variants—comprising rs429358 (*APOE*), rs2075650 (*TOMM40*), and rs12721046 (*APOC1*) SNPs to AD [31, 32]. We showed that a combination of the *APOE* ε4 allele (encoded by rs429358 minor allele) and minor alleles of rs2075650 and rs12721046 SNPs conferred a remarkably high risk of AD compared to the ε4-bearing compound genotypes which do not include minor alleles of rs2075650 and rs12721046 [32]. Here, we examine the associations of the *APOE* ε2 and ε4 alleles and the AD-risk-differentiating compound genotypes comprising rs429358, rs2075650, and rs12721046 SNPs with Aβ40, Aβ42, and tau AD biomarkers measured in CSF and plasma using data from three studies: the AD Neuroimaging Initiative (ADNI), the Atherosclerosis Risk in Communities (ARIC) study, and the Framingham Heart Study (FHS).

## RESULTS

### Study overview

We performed three types of analyses. First, we evaluated mean levels of Aβ40, Aβ42, and tau in CSF and plasma and the correlation between them. Second, we examined associations of the ε4 and ε2 alleles individually with AD biomarkers to establish benchmark effects in our samples. Third, as no significant associations of the ε2 allele with AD biomarkers were identified, we explored associations of the AD-risk- differentiating ε4-bearing compound genotypes comprising rs429358, rs2075650, and rs12721046 SNPs with the selected AD biomarkers. The goal was to identify whether the ε4 allele exerted effects on the selected biomarkers independently of the *TOMM40* rs2075650 and *APOC1* rs12721046 SNPs or whether its effect could be modulated by the latter two SNPs.

### Plasma and CSF AD biomarkers: mean levels and correlation

Table 1 and Supplementary Table 1 show that the mean levels of both baseline- and longitudinally-measured AD biomarkers vary across the study cohorts (see Methods), and they are substantially larger in CSF than in plasma. Correlation analysis of the AD biomarkers shows that CSF total tau and p-tau are perfectly correlated with the Pearson correlation coefficient *r* = 0.98 in ADNI (Supplementary Table 2). Given that p-tau was not available in other studies, the results for p-tau were not included.

**Table 1:** Baseline characteristics of the genotyped participants of European ancestry in the selected studies.

**Study**	**Source**	***N***	**Men (%)**	**Age (SD, SE), years**	**Aβ40 (SD, SE), pg/ml**	**Aβ42 (SD, SE), pg/ml**	**Tau (SD, SE), pg/ml**	**pTau (SD, SE), pg/ml**
**ARIC**	Plasma	1560	723 (46.3)	77.4 (5.4, 0.1)	247.0 (84.5, 2.1)	39.1 (11.2, 0.3)	NA	NA
**FHS_C1**	Plasma	636	227 (35.7)	79.8 (4.2, 0.2)	168.3 (41.9, 1.7)	45.4 (11.5, 0.5)	5.0 (1.5, 0.1)	NA
**FHS_C2**	Plasma	3095	1443 (46.6)	61.0 (9.5, 0.2)	159.6 (40.5, 0.8)	43.7 (10.3, 0.7)	4.2 (2.7, 0.1)	NA
**FHS_C3**	Plasma	3029	1424 (47.0)	45.7 (8.0, 0.1)	242.5 (58.0, 1.1)	42.7 (10.0, 0.2)	4.1 (1.5, 0.02)	NA
**ADNI-1**	Plasma	612	374 (61.1)	75.4 (6.7, 0.3)	152.9 (50.5, 2.0)	37.2 (11.3, 0.5)	2.9 (1.6, 0.1)	NA
**ADNI-1**	CSF	350	212 (60.6)	74.9 (7.1, 0.4)	7737.3 (2352.3, 125.7)	769.0 (352.0, 18.9)	302.3 (118.4, 6.3)	29.7 (13.6, 0.7)
**ADNI-2/GO**	CSF	360	203 (56.4)	72.9 (7.4, 0.4)	8590.2 (2437.7, 130.1)	920.6 (368.2, 19.4)	271.6 (118.6, 6.3)	25.7 (13.1, 0.7)

*N* denotes the number of subjects; Abbreviations: CSF: cerebrospinal fluid; SD: standard deviation; SE: standard error; NA: not available. ARIC is the Atherosclerosis Risk in Communities Study; FHS_C1, FHS_C2, and FHS_C3 denote the Framingham Heart Study parental, offspring, and grandchildren cohorts, respectively; ADNI-1 and ADNI-2/GO denote the Alzheimer’s disease Neuroimaging Initiative initial and extended cohorts, respectively. Age was defined at the time of the Alzheimer’s disease biomarker measurement.

### Associations of the *APOE* ε2 and ε4 alleles with AD biomarkers

The ε4 alleles were consistently associated (see Methods) with lower levels of both CSF and plasma Aβ42 in each cohort (Table 2 and Supplementary Table 3), despite the lack of significant correlation between them, *r* = 0.074 (*p* = 0.219) (Supplementary Table 2). Although CSF and plasma tau were weakly correlated (*r* = 0.155, *p* = 4.5 × 10^−3^), the *APOE* ε4 alleles were consistently associated with higher levels of CSF tau, but they were not significantly associated with plasma tau. The meta-analysis did not show significant associations with Aβ40 either in CSF or plasma, despite its modest-to-strong correlation with Aβ42, i.e., *r* = 0.388–0.703 in plasma and *r* = 0.253–0.304 in CSF. Qualitatively the same associations were observed using longitudinal measurements (Supplementary Table 3).

**Table 2:** Meta-analysis of the associations of the *APOE* ε4 allele with Alzheimer’s disease (AD) biomarkers.

**Biomarker**	**Source**	**N_reference_**	**N_ε4_**	**Beta**	**SE**	***P* value**	**Direction**
Aβ40	CSF^*^	334	304	−0.011	0.024	6.49E-01	−+????
Aβ40	Plasma^**^	5683	2109	−1.287	1.300	3.22E-01	−?−+−−
Aβ42	CSF^*^	259	292	−0.334	0.034	1.50E-22	−−????
Aβ42	Plasma^**^	5679	2106	−1.903	0.271	2.18E-12	−?−−−−
Tau	CSF^*^	341	302	0.261	0.031	6.58E-17	++????
Tau	Plasma^*^	3951	1484	0.006	0.011	5.67E-01	+?−+−?

N_reference_ denotes the number of carriers of the ε33 genotype used as a reference. N_ε4_ shows the number of the *APOE* ε4 carriers defined as having either ε34 or ε44 genotype. SE denotes standard error and CSF denotes cerebrospinal fluid. Asterisks denote a gamma general linear model with a log link function (^*^) and an ordinary linear model (^**^) used for the analysis. Column “Direction” shows sign of the effect beta in individual studies in the following order: ADNI-1, ADNI-2/GO, FHS_C1, FHS_C2, FHS_C3, and ARIC. Question mark indicates missing estimates. Note, CSF biomarkers were available only in ADNI cohorts; plasma biomarkers were not available in ADNI-2/GO, and tau was not reported in ARIC. Biomarkers were measured at baseline. More details with individual-study estimates are given Supplementary Table 3.

The ε2 alleles were associated only with CSF Aβ42 in the meta-analysis of the data available from baseline measurements in ADNI-1 and ADNI-2/GO (β = 0.143, *p* = 0.032) with a small number of subjects (*N* = 37), but not in longitudinal analysis of these data (β = 0.112, *p* = 0.280) with a larger number of observations (*N* = 80) (Supplementary Table 4).

### Associations of compound genotypes with AD biomarkers

Given associations of the ε4 alleles with Aβ42 and tau, we examined associations of the AD-risk differentiating compound genotypes comprising the ε4-encoding rs429358, *TOMM40* rs2075650, and *APOC1* rs12721046 SNPs with Aβ42 and tau measured at baseline (see Table 3 and Supplementary Table 5 for notations and the results). The ε4-bearing compound genotypes were significantly associated with lower levels of CSF and plasma Aβ42 and higher levels of CSF tau regardless of minor alleles of the other two SNPs. However, the compound genotype with the ε4 alleles and no minor alleles of the other two SNPs was associated with smaller levels of plasma tau (Table 3, 100+200), whereas no significant associations were observed for carriers of the compound genotypes aggregating minor alleles of those two SNPs (Table 3, 111+222 and 1XY+2XY). No significant associations were seen for non-carriers of the ε4 alleles who have minor alleles of rs2075650 and rs12721046 (Table 3, 0XY).

**Table 3:** Meta-analysis of the associations of compound genotypes with Alzheimer’s disease (AD) Aβ42 and tau biomarkers.

**Biomarker**	**Source**	**Genotype**	***N***	**Beta**	**SE**	***P* value**	**Direction**
Aβ42	CSF	0XY	59	0.026	0.055	6.37E-01	−+????
Aβ42	CSF	100+200	56	−0.272	0.057	2.13E-06	−−????
Aβ42	CSF	111+222	178	−0.341	0.039	2.46E-18	−−????
Aβ42	CSF	1XY+2XY	242	−0.373	0.037	2.18E-24	−−????
Aβ42	CSF	000	237	Reference			
Aβ42	Plasma	0XY	951	0.351	0.364	3.35E-01	−?−−++
Aβ42	Plasma	100+200	396	−2.305	0.551	2.82E-05	−?−−−−
Aβ42	Plasma	111+222	1462	−1.582	0.312	4.14E-07	−?−−−−
Aβ42	Plasma	1XY+2XY	1821	−1.656	0.287	7.98E-09	−?−−−−
Aβ42	Plasma	000	5549	Reference			
Tau	CSF	0XY	70	0.088	0.050	7.97E-02	++????
Tau	CSF	100+200	56	0.233	0.056	2.85E-05	++????
Tau	CSF	111+222	189	0.293	0.035	4.87E-17	++????
Tau	CSF	1XY+2XY	253	0.294	0.032	6.75E-20	++????
Tau	CSF	000	326	Reference			
Tau	Plasma	0XY	647	0.009	0.013	4.91E-01	−?−+−?
Tau	Plasma	100+200	289	−0.045	0.018	1.28E-02	+?−−−?
Tau	Plasma	111+222	1033	−0.010	0.011	3.66E-01	+?−−−?
Tau	Plasma	1XY+2XY	1280	−0.002	0.010	8.42E-01	+?−−−?
Tau	Plasma	000	3913	Reference			

Column “Genotype” shows compound genotypes encoded by triples of numbers and X and Y letters. Numbers show the counts of minor alleles (i.e., 0, 1, 2) in rs429358_T/c, rs2075650_A/g or rs12721046_G/a SNP, in that order. The upper/lower case denotes here major/minor allele. The most frequent 000 genotype denotes the major allele homozygote for all three SNPs, i.e., rs429358_TT, rs2075650_AA, rs12721046_GG. The 100+200 genotype indicates rs429358_Tc, rs2075650_AA, rs12721046_GG (100) and rs429358_cc, rs2075650_AA, rs12721046_GG (200). The 111+222 genotype denotes rs429358_Tc, rs2075650_Ag, rs12721046_Ga (111) and rs429358_cc, rs2075650_gg, rs12721046_aa (222). Letters X and Y indicate aggregation of minor alleles of rs2075650 and rs12721046, respectively. Then, 0XY aggregates all non-ε4 genotypes except 000. The 1XY+2XY genotype aggregates rs429358_Tc (1) and rs429358_cc (2) and all genotypes of rs2075650 (X) and rs12721046 (Y), except major allele homozygote of both SNPs, rs2075650_AA and rs12721046_GG (00), because it is included in the 100+200 genotype. Column “Direction” shows sign of the effect beta in individual studies in the following order: ADNI-1, ADNI-2/GO, FHS_C1, FHS_C2, FHS_C3, and ARIC. Question mark indicates missing estimates. Note, cerebrospinal fluid (CSF) biomarkers were available only in ADNI cohorts; plasma biomarkers were not available in ADNI-2/GO, and tau was not reported in ARIC. A gamma general linear model with a log link function was used for all biomarkers except Aβ42 measured in plasma. SE denotes standard error. More details with individual-study estimates are given Supplementary Table 5.

The ε4-bearing compound genotypes having (111+222 and 1XY+2XY) and not having (100+200) minor alleles of rs2075650 and rs12721046 represent polygenic profiles conferring higher and lower AD risk, respectively [32]. Then, we quantified potential differences in the associations of these compound genotypes with Aβ42 and tau (Table 4 and Supplementary Table 6). No significant differences in the associations of these ε4-bearing higher and lower AD risk compound genotypes with CSF and plasma Aβ42 were identified. Carrying the ε4 allele and minor alleles of rs2075650 and rs12721046 was associated with significantly higher levels of plasma tau compared to having the ε4 allele and no minor alleles of these two SNPs, β = 0.047, *p* = 0.023 (Table 4, 1XY+2XY), consistently across all cohorts (Supplementary Table 6). The same effect direction was also observed for CSF tau in the meta-analysis, β = 0.060, *p* = 0.270, and each cohort (Supplementary Table 6), although the estimates did not attain the significance due to a 5-fold smaller sample with CSF tau than plasma tau. Because plasma and CSF tau were measured in ADNI-1, we were able to examine the association of the aggregated compound genotype 1XY+2XY with CSF tau with adjustment for plasma tau. This analysis did not show the mediating effect of plasma tau because the difference in the associations between the adjusted (β = 0.026, *p* = 0.700) and unadjusted (β = 0.030, *p* = 0.650) models by plasma tau in ADNI-1 was trivial.

**Table 4:** Comparative meta-analysis of the associations of the selected compound genotypes with Alzheimer’s disease (AD) Aβ42 and tau biomarkers.

**Biomarker**	**Source**	**Genotype**	***N***	**Beta**	**SE**	***P* value**	**Direction**
Aβ42	CSF	111+222	178	-0.054	0.062	3.77E-01	−−????
Aβ42	CSF	1XY+2XY	242	-0.091	0.060	1.32E-01	−−????
Aβ42	CSF	100+200	56	Reference			
Aβ42	Plasma	111+222	1462	0.619	0.569	2.77E-01	−?++++
Aβ42	Plasma	1XY+2XY	1821	0.509	0.553	3.57E-01	−?++++
Aβ42	Plasma	100+200	396	Reference			
Tau	CSF	111+222	189	0.066	0.057	2.46E-01	++????
Tau	CSF	1XY+2XY	253	0.060	0.054	2.70E-01	++????
Tau	CSF	100+200	56	Reference			
Tau	Plasma	111+222	1033	0.033	0.021	1.25E-01	+?+++?
Tau	Plasma	1XY+2XY	1280	0.047	0.021	2.27E-02	+?+++?
Tau	Plasma	100+200	289	Reference			

Column “Genotype” shows compound genotypes encoded by triples of numbers and X and Y letters; these notations are detailed in Table 3 footnote. Column “Direction” shows sign of the effect beta in individual studies in the same order as in Table 3. A gamma general linear model with a log link function was used for all biomarkers except Aβ42 measured in plasma. SE denotes standard error. CSF denotes cerebrospinal fluid. More details with individual-study estimates are given Supplementary Table 6.

We found that excluding carriers of the ε2 alleles did not make a difference (Supplementary Table 7).

## DISCUSSION

We performed the analysis of the associations of the *APOE* ε2 and ε4 alleles and polygenic profiles represented by combinations of variants of the ε4 encoding rs429358, *TOMM40* rs2075650, and *APOC1* rs12721046 SNPs with CSF and plasma Aβ40, Aβ42, and tau AD biomarkers. Our primary finding is that the ε4-bearing polygenic profiles conferring higher and lower AD risks are differently associated with tau but not Aβ42. The other main results of our work are characterizations of the associations of the *APOE* ε2 and ε4 alleles with Aβ40, Aβ42, and tau biomarkers in ADNI-1, ADNI-2/GO, ARIC, and three FHS cohorts.

### *APOE* ε2 and ε4 alleles and AD biomarkers

Our analysis confirmed robust associations of the ε4 alleles with both plasma and CSF Aβ42 levels [17] (Table 2). Unlike Aβ42, no significant associations of the ε4 allele with Aβ40 were identified despite a relatively high correlation between these biomarkers. By showing the robust and highly significant associations of the ε4 allele with CSF tau, our analysis supports previous findings on the connections between the ε4 allele and tau aggregation [17, 33, 34]. Meanwhile, we report no significant associations of the ε4 allele with plasma tau (Table 2).

We show that the ε2 allele is associated with CSF Aβ42 at nominal significance in the ADNI sample, which corroborates the results of previous analysis in this sample [35]. Nevertheless, longitudinal analysis using a larger number of CSF Aβ42 measurements from multiple visits did not confirm the significance of this association. However, ADNI sample includes relatively old subjects (Table 1), and longitudinal assessment was done at even older ages (Supplementary Table 1). Because studies showed that the ε2 allele might not be associated with CSF Aβ42 at older ages [36], the addition of Aβ42 measurements at older ages affected the significance of the estimate in our study. The ε2 allele was not significantly associated with plasma Aβ42 and CSF and plasma Aβ40 and tau (Supplementary Table 4).

### Polygenic profiles and AD biomarkers

Our prior analysis identified that the ε4-bearing compound genotypes examined in the current study of AD biomarkers exerted 89% (OR [odds ratio] = 1.89, *p* = 4.69 × 10^−13^) higher odds of AD when the ε4 alleles clustered with minor alleles of *TOMM40* rs2075650 and *APOC1* rs12721046 SNPs than major alleles of these two SNPs, i.e., when the 1XY+2XY compound genotype (carriers of the ε4 alleles who also carry minor alleles of rs2075650 and rs12721046) was contrasted by 100+200 genotype (carriers of the ε4 alleles who do not carry minor alleles of rs2075650 and rs12721046) [32].

In this study, we show no significant difference in the associations of the 1XY+2XY and 100+200 compound genotypes with either plasma or CSF Aβ42. This result implies that the association of the ε4 allele with Aβ42 is likely due to this allele itself because its association is not significantly modulated by minor alleles of rs2075650 and rs12721046. In contrast, a significant difference in the associations of the 1XY+2XY and 100+200 compound genotypes with plasma tau (Table 4) (and, potentially, with CSF tau, Supplementary Table 6) suggests joint roles of the ε4 allele and minor alleles of rs2075650 and rs12721046 in tau aggregation. Because the 1XY+2XY compound genotype entails 89% higher odds of AD than the 100+200 genotype, the 1XY+2XY genotype is tighter linked to neurodegeneration than 100+200. Therefore, the identified difference in the associations of the ε4-bearing polygenic profiles conferring higher and lower AD risks is tied to tau but not Aβ.

### Insights on potential *APOE*-related mechanism of AD

**Figure 1:**
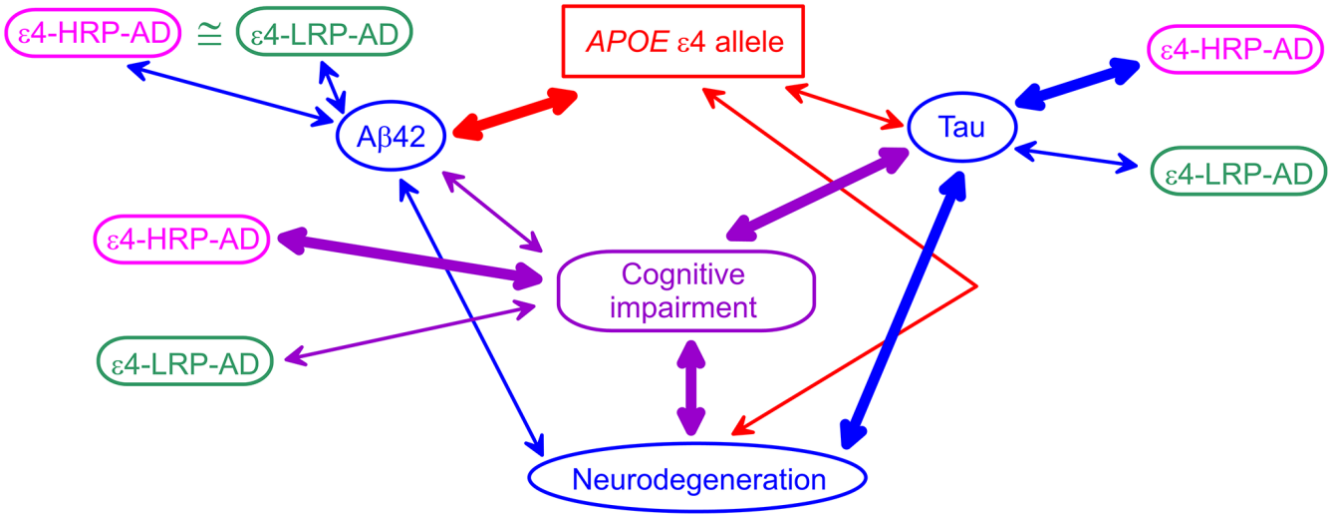
**A schematic diagram of potential *APOE*-related mechanism of Alzheimer’s disease (AD).** Blue ovals indicate AD biomarkers. The red rectangle shows the *APOE* ε4 allele. The purple rounded rectangle indicates cognitive impairment (AD or mild cognitive impairment). Magenta and green rounded rectangles denote the ε4-bearing higher-AD-risk profile (ε4-HRP-AD) and lower-AD-risk profile (ε4-LRP-AD), respectively. The thickness of the arrows denotes tighter (thick lines) and weaker (thin lines) links between genetic variants, AD biomarkers, and cognitive impairment.

Our results align with prior findings based on the associations of the ε4 alleles with AD biomarkers. Differential associations of the ε4-bearing polygenic profiles with AD biomarkers help clarify connections of the ε4 allele with AD biomarkers and the role of Aβ and tau pathologies in AD pathogenesis. Indeed, studies showed that Aβ42 was tighter linked with the ε4 allele than clinically diagnosed cognitive impairment (AD or MCI), whereas tau and neurodegeneration were stronger associated with cognitive impairment than the ε4 alleles [15] (Figure 1). The CSF Aβ42 appears to be independently associated with AD and the ε4 alleles, as was shown in [16] and corroborated in [37]. PET imaging showed a tighter linkage of neurodegeneration to tau pathology than Aβ pathology [38]. These findings implicate the role of the ε4 alleles in AD via both Aβ and tau pathologies. They emphasize the primary role of the ε4 alleles in Aβ pathology, the reduced role of this allele in tau pathology, and the stronger link of tau with neurodegeneration. These findings support the mechanism that Aβ pathology is pronounced before the emergence of the AD clinical manifestation, whereas AD manifestation develops due to neurodegeneration [12, 15, 39]. Then, our results are aligned with these findings because: (i) the polygenic profile comprising the ε4 allele and minor alleles of rs2075650 and rs12721046 is a proxy for cognitive impairment because it is tied to higher AD risk (ε4-HRP-AD), (ii) this higher-AD-risk profile is differentiated from the lower-AD-risk profile (ε4-LRP-AD) based on the associations with tau, and (iii) this profile is not differentiated based on the associations with Aβ42. These insights indicate that the ε4 allele plays a role in Aβ pathology, whereas its role in tau pathology is modulated by minor alleles of *TOMM40* rs2075650 and *APOC1* rs12721046 SNPs when they are clustered in the higher-AD-risk profile. Therefore, our findings suggest that modulation of the effect of the ε4 allele by *TOMM40* and *APOC1* variants indicates a potential genetic mechanism of differential roles of Aβ and tau in AD pathogenesis.

### Limitations

We acknowledge the limitations of this study. First, the samples used in this analysis were not optimal to examine the roles of compound genotypes comprising minor allele homozygotes of rs429358, rs2075650, and rs12721046 SNPs. Second, we did not explore the potential roles of haplotypes containing these SNPs due to the limited number of minor allele homozygotes. Third, further analyses using larger samples are needed to robustly examine associations of compound genotypes with CSF Aβ and tau. Fourth, we did not look at the potential roles of sex due to the limited sample size, particularly for CSF measurements.

## MATERIALS AND METHODS

### Study cohorts

The data for this paper were from the ADNI initial (ADNI-1) and extended (ADNI-2/GO) cohorts [40, 41], the ARIC study [42], and the FHS parental (FHS_C1), offspring (FHS_C2), and grandchildren (FHS_C3) cohorts [43]. The basic characteristics of the available samples are given in Table 1.

### AD biomarkers

#### Alzheimer's disease neuroimaging initiative

Data used in the preparation of this article were obtained from the ADNI database (https://adni.loni.usc.edu/). The ADNI was launched in 2003 as a public-private partnership, led by Principal Investigator Michael W. Weiner, MD. The primary goal of ADNI has been to test whether serial magnetic resonance imaging (MRI), PET, other biological markers, and clinical and neuropsychological assessment can be combined to measure the progression of mild cognitive impairment (MCI) and early AD. For up-to-date information, see https://adni.loni.usc.edu/.

Concentrations of Aβ42 and Aβ40 in plasma were measured using the Luminex xMAP platform and Innogenetics INNO-BIA AlzBio3 immunoassay reagents (Innogenetics NV, Ghent, Belgium). Plasma tau was measured in ADNI-1 and it was analyzed by the Single-Molecule array (Simoa) technique and the Human total tau assay. Concentrations of Aβ42, tau, and p-tau181 (p-tau) in CSF were assessed using an automated Elecsys cobas e 601 tool based on Roche Elecsys immunoassays [44]. CSF samples were collected in ADNI-1 and ADNI-2/GO between 2005 and 2016. In addition, immunoassay-independent measurements of CSF Aβ (Aβ40 and Aβ42) were done using a candidate reference 2D-UPLC/tandem mass spectrometry method.

#### Atherosclerosis risk in communities study

Plasma Aβ40 and Aβ42 concentrations were measured at the 5th examination. Amyloid was quantified by the Department of Molecular Pharmacology and Experimental Therapeutics at Mayo Clinic (Jacksonville, FL) from August to December 2014 using the INNO- BIA assay (Innogenetics NV, Ghent, Belgium). The Luminex 200 IS Total system was used for detecting fluorescence emitted by beads (xMAP microspheres; conjugate 1A) that were bound to Aβ40 and Aβ42 [45]. The minimal detectable levels for Aβ42 and Aβ40 were 12 pg/ml and 15 pg/ml, respectively [46, 47].

#### Framingham heart study

Plasma Aβ42 and Aβ40 concentrations were measured at the 23rd examination in FHS_C1, the 7th examination in FHS_C2, and the 2nd examination in FHS_C3 cohorts. Amyloid was quantified by the same Mayo Clinic facility as for ARIC from June to August 2012 and from January to June 2014 using the same Innogenetics NV assays. The minimal detectable levels were 12pg/ml for Aβ40 and 5pg/ml for Aβ42. Plasma tau was measured in blood samples obtained at the 28th examination in FHS_C1, the 8th examination in FHS_C2, and the 2nd examination in FHS_C3. Plasma samples were assayed from February to March 2017. Total tau was measured by Quanterix (Lexington, MA, USA) using a Simoa™ tau 2.0 Kit and a Simoa HD-1 analyzer. This is a molecule enzyme-linked immunosorbent assay (digital ELISA) with a minimum detectable level of 0.019 pg/ml [48].

### Genotypes

We examined associations of the *APOE* ε4 and ε2 alleles encoded by minor alleles of rs429358 (T/c; upper/lower case denotes here major/minor allele) and rs7412 (C/t), respectively, and compound genotypes comprising rs429358, rs2075650 (*TOMM40*, A/g), and rs12721046 (*APOC1*, G/a) SNPs. To maximize the sample size, missing genotypes for some subjects in each study were imputed (Michigan Imputation Server, HRC panel). We retained genotypes with high imputation quality (*r*^2^ > 0.8).

The ε4 allele was defined in the absence of the ε2 allele, i.e., by ε3/ε4 and ε4/ε4 genotypes. Likewise, the ε2 allele was defined in the absence of the ε4 allele, i.e., by ε2/ε3 and ε2/ε2 genotypes. The triple of rs429358, rs2075650, and rs12721046 SNPs, which are in about the same moderate pair-wise linkage disequilibrium *r*^2^ ≈ 0.49, was selected because its minor allele compound genotypes conferred an exceptionally high risk of AD [31, 32]. To streamline notations for triples, we used definitions based on the counts of minor alleles (i.e., 0, 1, 2) in an SNP, as detailed in Table 3 footnotes.

### Statistical analysis

The associations of the ε4 or ε2 allele and compound genotypes of interest with AD biomarkers were evaluated using their baseline (Table 1) and longitudinal (Supplementary Table 1) measurements. We used the ε3/ε3 genotype as a reference in the analyses of the ε4 or ε2 allele. References in the analyses of compound genotypes varied and are shown in the corresponding tables. AD biomarkers were used as outcomes. We employed the glm and lm functions from base R, as well as the glmer and lmer functions from the lme4 package [49]. Biomarkers CSF Aβ40, Aβ42, tau, and p-tau, and plasma tau showed gamma-like distributions; therefore, functions glm and glmer were used to run gamma general linear models with a log link function. The regression coefficients beta in these models can be interpreted as a fraction (or percentage) of change of a continuous outcome. All models were adjusted for sex, age, and age squared. If the model had convergence issues, age squared was removed. The analysis using longitudinal measurements was run in ADNI, and required the use of lmer and glmer to account for the correlation between repeated measurements on the individual. In FHS lmer and glmer were used to account for familial correlation. To examine the potential role of the ε2 alleles, we also performed the analysis excluding all subjects with ε2 alleles. No other adjustments were made. Meta-analysis was performed using a fixed-effects model with inverse-variance weighting. We used *p* < 0.05 as a significance level.

## Supplementary Materials

Supplementary Materials

Supplementary Tables
