# Supplementary material for: Associations of the APOE ε2 and ε4 alleles and polygenic profiles comprising APOE-TOMM40-APOC1 variants with Alzheimer’s disease biomarkers
Source: Aging (Albany NY). 2022 Nov 17;14(24):9782–804. doi: 10.18632/aging.204384 (PMC9831745; doi:10.18632/aging.204384)
Supplement: Supplementary Materials [file aging-14-204384-s001.pdf]

SUPPLEMENTARY MATERIALS

This article was prepared using a data obtained through dbGaP (accession numbers phs000007.v31 [FHS] and phs000280, v.7 [ARIC]) and ADNI data obtained through the Image and Data Archive (IDA) run by the Laboratory of Neuro Imaging (LONI).

The Framingham Heart Study (FHS) is conducted and supported by the National Heart, Lung, and Blood Institute (NHLBI) in collaboration with Boston University (Contract No. N01-HC-25195 and HHSN268201500001I). This manuscript was not prepared in collaboration with investigators of the FHS and does not necessarily reflect the opinions or views of the FHS, Boston University, or NHLBI. Funding for SHARe Affymetrix genotyping was provided by NHLBI Contract N02-HL-64278. SHARe Illumina genotyping was provided under an agreement between Illumina and Boston University. Funding for CARe genotyping was provided by NHLBI Contract N01-HC-65226. Funding support for the Framingham Dementia dataset was provided by NIH/NIA grant R01 AG08122.

The Atherosclerosis Risk in Communities (ARIC) study has been funded in whole or in part with Federal funds from the National Heart, Lung, and Blood Institute, National Institute of Health, Department of Health and Human Services, under contract numbers (HHSN268201700001I, HHSN268201700002I, HHSN268201700003I, HHSN268201700004I, and HHSN268201700005I). The authors thank the staff and participants of the ARIC study for their important contributions. Funding for CARe genotyping was provided by NHLBI Contract N01-HC-65226.Funding for GENEVA was provided by National Human Genome Research Institute grant U01HG004402 (E. Boerwinkle). The datasets used for the analyses in this manuscript were obtained from dbGaP through dbGaP accession study number phs000280, v.7.

The Alzheimer's Disease Neuroimaging Initiative (ADNI). Data collection and sharing was funded by the ADNI (National Institutes of Health Grant U01 AG024904) and DOD ADNI (Department of Defense award number W81XWH-12-2-0012). ADNI is funded by the National Institute on Aging, the National Institute of Biomedical Imaging and Bioengineering, and through generous contributions from the following: AbbVie, Alzheimer's Association; Alzheimer's Drug Discovery Foundation; Araclon Biotech; BioClinica, Inc.; Biogen; Bristol-Myers Squibb Company; CereSpir, Inc.; Cogstate; Eisai Inc.; Elan Pharmaceuticals, Inc.; Eli Lilly and Company; EuroImmun; F. Hoffmann-La Roche Ltd and its affiliated company Genentech, Inc.; Fujirebio; GE Healthcare; IXICO Ltd.; Janssen Alzheimer Immunotherapy Research and Development, LLC.; Johnson and Johnson Pharmaceutical Research and Development LLC.; Lumosity; Lundbeck; Merck and Co., Inc.; Meso Scale Diagnostics, LLC.; NeuroRx Research; Neurotrack Technologies; Novartis Pharmaceuticals Corporation; Pfizer Inc.; Piramal Imaging; Servier; Takeda Pharmaceutical Company; and Transition Therapeutics. The Canadian Institutes of Health Research is providing funds to support ADNI clinical sites in Canada. Private sector contributions are facilitated by the Foundation for the National Institutes of Health (www.fnih.org). The grantee organization is the Northern California Institute for Research and Education, and the study is coordinated by the Alzheimer's Therapeutic Research Institute at the University of Southern California. ADNI data are disseminated by the Laboratory for Neuro Imaging at the University of Southern California.
