# Supplementary material for: Associations of the APOE ε2 and ε4 alleles and polygenic profiles comprising APOE-TOMM40-APOC1 variants with Alzheimer’s disease biomarkers
Source: Aging (Albany NY). 2022 Nov 17;14(24):9782–804. doi: 10.18632/aging.204384 (PMC9831745; doi:10.18632/aging.204384)
Supplement: Supplementary Tables [file aging-14-204384-s002.pdf]

SUPPLEMENTARY TABLES

Supplementary Table 1. Basic characteristics of the genotyped participants of European ancestry in the selected studies over all available longitudinal measurements.

Study	Source	*N*	Men (%)	Age (SD, SE), years	Aβ40 (SD, SE), pg/ml	Aβ42 (SD, SE), pg/ml	Tau (SD, SE), pg/ml	pTau (SD, SE), pg/ml
**ADNI-1**	Plasma	2077	1269 (61.1)	76.6 (6.6, 0.1)	164.4 (46.7, 1.0)	39.9 (10.9, 0.2)	NA	NA
**ADNI-1**	CSF	948	571 (60.2)	76.7 (6.9, 0.2)	7844.7 (2326.7, 97.1)	768.6 (365.6, 11.9)	307.6 (122.3, 4.0)	29.7 (13.7, 0.4)
**ADNI-2/GO**	CSF	636	357 (56.1)	73.9 (7.4, 0.3)	8652.0 (2492.9, 105.0)	901.4 (369.0, 14.6)	279.6 (121.9, 4.8)	26.3 (13.3, 0.5)

Abbreviation: CSF: cerebrospinal fluid; SD: standard deviation; SE: standard error. *N* denotes the total number of observations of Alzheimer's disease (AD) biomarkers at four (plasma) and up to eight (CSF) examinations. ADNI-1 and ADNI-2/GO denote the AD Neuroimaging Initiative initial and extended cohorts, respectively. Age was defined at the time of the AD biomarker measurement.

Supplementary Table 2. Pearson pair-wise correlation estimates between Alzheimer's disease (AD) biomarkers in selected studies.

Study	Biomarker_1	Biomarker_2	*N*	Correlation, *r*	*P* value
ADNI-1	Aβ40_plasma	Aβ42_plasma	608	0.703	6.80E-92
ADNI-1	Aβ40_plasma	Tau_plasma	449	0.127	7.03E-03
ADNI-1	Aβ42_plasma	Tau_plasma	450	0.064	1.73E-01
ADNI-1	Aβ40_CSF	Aβ42_CSF	305	0.304	6.41E-08
ADNI-1	Aβ40_CSF	Tau_CSF	339	0.524	2.85E-25
ADNI-1	Aβ40_CSF	pTau_CSF	339	0.460	4.01E-19
ADNI-1	Aβ42_CSF	Tau_CSF	308	−0.275	9.27E-07
ADNI-1	Aβ42_CSF	pTau_CSF	308	−0.314	1.78E-08
ADNI-1	Tau_CSF	pTau_CSF	345	0.980	9.17E-244
ADNI-1	Aβ42_plasma	Aβ42_CSF	280	0.074	2.19E-01
ADNI-1	Aβ40_plasma	Aβ40_CSF	313	0.005	9.24E-01
ADNI-1	Tau_CSF	Tau_plasma	334	0.155	4.51E-03
ADNI-2/GO	Aβ40_CSF	Aβ42_CSF	274	0.253	2.22E-05
ADNI-2/GO	Aβ40_CSF	Tau_CSF	351	0.542	3.78E-28
ADNI-2/GO	Aβ40_CSF	pTau_CSF	351	0.464	4.13E-20
ADNI-2/GO	Aβ42_CSF	Tau_CSF	283	−0.269	4.39E-06
ADNI-2/GO	Aβ42_CSF	pTau_CSF	283	−0.326	2.04E-08
ADNI-2/GO	Tau_CSF	pTau_CSF	360	0.979	3.03E-250
FHS_C1	Aβ40_plasma	Aβ42_plasma	636	0.613	7.16E-67
FHS_C1	Aβ40_plasma	Tau_plasma	128	0.103	2.49E-01
FHS_C1	Aβ42_plasma	Tau_plasma	128	0.096	2.80E-01
FHS_C2	Aβ40_plasma	Aβ42_plasma	3095	0.500	9.43E-196
FHS_C2	Aβ40_plasma	Tau_plasma	2554	0.108	5.04E-08
FHS_C2	Aβ42_plasma	Tau_plasma	2554	0.068	5.77E-04
FHS_C3	Aβ40_plasma	Aβ42_plasma	3029	0.388	1.40E-109
FHS_C3	Aβ40_plasma	Tau_plasma	3026	0.021	2.45E-01
FHS_C3	Aβ42_plasma	Tau_plasma	3026	0.043	1.91E-02

N denotes the number of subjects. Abbreviation: CSF: cerebrospinal fluid. FHS_C1, FHS_C2, and FHS_C3 denote the Framingham Heart Study parental, offspring, and grandchildren cohorts, respectively; ADNI-1 and ADNI-2/GO denote the AD Neuroimaging Initiative initial and extended cohorts, respectively.

Supplementary Table 3. Associations of the APOE ε4 allele with Alzheimer's disease (AD) biomarkers.

Study	Type	Biomarker	Source	Genotype	*N* subjects	*N* observations	Beta	SE	*P* value
ADNI-1	Baseline	Aβ40	CSF	e4	171	171	−0.047	0.034	1.76E-01
ADNI-1	Baseline	Aβ40	CSF	e33	149	149	Reference		
ADNI-2/GO	Baseline	Aβ40	CSF	e4	133	133	0.022	0.033	5.06E-01
ADNI-2/GO	Baseline	Aβ40	CSF	e33	185	185	Reference		
Meta	Baseline	Aβ40	CSF	e4	304	304	−0.011	0.024	6.49E-01
Meta	Baseline	Aβ40	CSF	e33	334	334	Reference		
ADNI-1	Longitudinal	Aβ40	CSF	e4	173	271	−0.051	0.049	2.97E-01
ADNI-1	Longitudinal	Aβ40	CSF	e33	152	267	Reference		
ADNI-2/GO	Longitudinal	Aβ40	CSF	e4	134	216	0.034	0.045	4.58E-01
ADNI-2/GO	Longitudinal	Aβ40	CSF	e33	186	290	Reference		
Meta	Longitudinal	Aβ40	CSF	e4	307	487	−0.005	0.033	8.70E-01
Meta	Longitudinal	Aβ40	CSF	e33	338	557	Reference		
ADNI-1	Baseline	Aβ40	Plasma	e4	291	291	−0.795	4.345	8.55E-01
ADNI-1	Baseline	Aβ40	Plasma	e33	270	270	Reference		
FHS_C1	Baseline	Aβ40	Plasma	e4	114	114	−8.986	4.397	4.15E-02
FHS_C1	Baseline	Aβ40	Plasma	e33	432	432	Reference		
FHS_C2	Baseline	Aβ40	Plasma	e4	630	630	0.666	1.827	7.16E-01
FHS_C2	Baseline	Aβ40	Plasma	e33	1982	1982	Reference		
FHS_C3	Baseline	Aβ40	Plasma	e4	674	674	−2.299	2.609	3.78E-01
FHS_C3	Baseline	Aβ40	Plasma	e33	1916	1916	Reference		
ARIC	Baseline	Aβ40	Plasma	e4	400	400	−2.837	4.938	5.66E-01
ARIC	Baseline	Aβ40	Plasma	e33	1083	1083	Reference		
Meta	Baseline	Aβ40	Plasma	e4	2109	2109	−1.287	1.300	3.22E-01
Meta	Baseline	Aβ40	Plasma	e33	5683	5683	Reference		
ADNI-1	Longitudinal	Aβ40	Plasma	e4	296	961	−1.237	3.270	7.05E-01
ADNI-1	Longitudinal	Aβ40	Plasma	e33	277	942	Reference		
Meta	Longitudinal	Aβ40	Plasma	e4	296	961	−1.237	3.270	7.05E-01
Meta	Longitudinal	Aβ40	Plasma	e33	277	942	Reference		
ADNI-1	Baseline	Aβ42	CSF	e4	165	165	−0.407	0.048	7.15E-16
ADNI-1	Baseline	Aβ42	CSF	e33	125	125	Reference		
ADNI-2/GO	Baseline	Aβ42	CSF	e4	127	127	−0.256	0.049	3.94E-07
ADNI-2/GO	Baseline	Aβ42	CSF	e33	134	134	Reference		
Meta	Baseline	Aβ42	CSF	e4	292	292	−0.334	0.034	1.50E-22
Meta	Baseline	Aβ42	CSF	e33	259	259	Reference		
ADNI-1	Longitudinal	Aβ42	CSF	e4	178	433	−0.478	0.062	4.85E-14
ADNI-1	Longitudinal	Aβ42	CSF	e33	151	374	Reference		
ADNI-2/GO	Longitudinal	Aβ42	CSF	e4	132	223	−0.274	0.069	9.49E-05
ADNI-2/GO	Longitudinal	Aβ42	CSF	e33	142	231	Reference		
Meta	Longitudinal	Aβ42	CSF	e4	310	656	−0.387	0.046	7.32E-17
Meta	Longitudinal	Aβ42	CSF	e33	293	605	Reference		
ADNI-1	Baseline	Aβ42	Plasma	e4	291	291	−1.987	0.944	3.57E-02
ADNI-1	Baseline	Aβ42	Plasma	e33	273	273	Reference		
FHS_C1	Baseline	Aβ42	Plasma	e4	114	114	−3.912	1.194	1.12E-03

FHS_C1	Baseline	Aβ42	Plasma	e33	432	432	Reference		
FHS_C2	Baseline	Aβ42	Plasma	e4	630	630	−1.891	0.466	5.04E-05
FHS_C2	Baseline	Aβ42	Plasma	e33	1982	1982	Reference		
FHS_C3	Baseline	Aβ42	Plasma	e4	674	674	−1.019	0.454	2.50E-02
FHS_C3	Baseline	Aβ42	Plasma	e33	1916	1916	Reference		
ARIC	Baseline	Aβ42	Plasma	e4	397	397	−3.114	0.654	2.10E-06
ARIC	Baseline	Aβ42	Plasma	e33	1076	1076	Reference		
Meta	Baseline	Aβ42	Plasma	e4	2106	2106	−1.903	0.271	2.18E-12
Meta	Baseline	Aβ42	Plasma	e33	5679	5679	Reference		
ADNI-1	Longitudinal	Aβ42	Plasma	e4	296	962	−1.995	0.799	1.26E-02
ADNI-1	Longitudinal	Aβ42	Plasma	e33	277	950	Reference		
Meta	Longitudinal	Aβ42	Plasma	e4	296	962	−1.995	0.799	1.26E-02
Meta	Longitudinal	Aβ42	Plasma	e33	277	950	Reference		
ADNI-1	Baseline	Tau	CSF	e4	167	167	0.204	0.043	3.04E-06
ADNI-1	Baseline	Tau	CSF	e33	149	149	Reference		
ADNI-2/GO	Baseline	Tau	CSF	e4	135	135	0.323	0.045	6.80E-12
ADNI-2/GO	Baseline	Tau	CSF	e33	192	192	Reference		
Meta	Baseline	Tau	CSF	e4	302	302	0.261	0.031	6.58E-17
Meta	Baseline	Tau	CSF	e33	341	341	Reference		
ADNI-1	Longitudinal	Tau	CSF	e4	179	435	0.217	0.059	2.36E-04
ADNI-1	Longitudinal	Tau	CSF	e33	165	433	Reference		
ADNI-2/GO	Longitudinal	Tau	CSF	e4	138	237	0.304	0.059	3.13E-07
ADNI-2/GO	Longitudinal	Tau	CSF	e33	194	334	Reference		
Meta	Longitudinal	Tau	CSF	e4	317	672	0.260	0.042	3.59E-10
Meta	Longitudinal	Tau	CSF	e33	359	767	Reference		
ADNI-1	Baseline	Tau	Plasma	e4	235	235	0.102	0.053	5.75E-02
ADNI-1	Baseline	Tau	Plasma	e33	223	223	Reference		
FHS_C1	Baseline	Tau	Plasma	e4	20	20	−0.113	0.082	1.72E-01
FHS_C1	Baseline	Tau	Plasma	e33	93	93	Reference		
FHS_C2	Baseline	Tau	Plasma	e4	556	556	0.019	0.019	3.25E-01
FHS_C2	Baseline	Tau	Plasma	e33	1721	1721	Reference		
FHS_C3	Baseline	Tau	Plasma	e4	673	673	−0.003	0.013	8.31E-01
FHS_C3	Baseline	Tau	Plasma	e33	1914	1914	Reference		
Meta	Baseline	Tau	Plasma	e4	1484	1484	0.006	0.011	5.67E-01
Meta	Baseline	Tau	Plasma	e33	3951	3951	Reference		

ARIC is the Atherosclerosis Risk in Communities Study. FHS_C1, FHS_C2, and FHS_C3 denote the Framingham Heart Study parental, offspring, and grandchildren cohorts, respectively. ADNI-1 and ADNI-2/GO denote the Alzheimer's disease Neuroimaging Initiative initial and extended cohorts, respectively. Meta shows the results of meta-analysis. Meta-analysis field was shown for consistency for each biomarker even if the analysis was performed in one study only. Column "Type" indicates biomarker measurements at baseline or longitudinally. Abbreviations: CSF: cerebrospinal fluid; SE: standard error. The APOE ε4 allele was defined as e34 and e44 genotypes. A gamma general linear model with a log link function was used for all biomarkers except Aβ40 and Aβ42 measured in plasma.

Supplementary Table 4. Associations of the APOE ε2 allele with Alzheimer's disease (AD) biomarkers.

Study	Type	Biomarker	Source	Genotype	*N* subjects	*N* observations	Beta	SE	*P* value
ADNI-1	Baseline	Aβ40	CSF	e4	25	25	0.063	0.069	3.62E-01
ADNI-1	Baseline	Aβ40	CSF	e33	149	149	Reference		
ADNI-2/GO	Baseline	Aβ40	CSF	e4	31	31	−0.032	0.055	5.68E-01
ADNI-2/GO	Baseline	Aβ40	CSF	e33	185	185	Reference		
Meta	Baseline	Aβ40	CSF	e4	56	56	0.006	0.043	8.99E-01
Meta	Baseline	Aβ40	CSF	e33	334	334	Reference		
ADNI-1	Longitudinal	Aβ40	CSF	e4	25	27	0.075	0.093	4.21E-01
ADNI-1	Longitudinal	Aβ40	CSF	e33	152	267	Reference		
ADNI-2/GO	Longitudinal	Aβ40	CSF	e4	31	54	−0.058	0.080	4.67E-01
ADNI-2/GO	Longitudinal	Aβ40	CSF	e33	186	290	Reference		
Meta	Longitudinal	Aβ40	CSF	e4	56	81	−0.002	0.060	9.80E-01
Meta	Longitudinal	Aβ40	CSF	e33	338	557	Reference		
ADNI-1	Baseline	Aβ40	Plasma	e4	36	36	0.679	8.602	9.37E-01
ADNI-1	Baseline	Aβ40	Plasma	e33	270	270	Reference		
FHS_C1	Baseline	Aβ40	Plasma	e4	74	74	−1.922	5.561	7.30E-01
FHS_C1	Baseline	Aβ40	Plasma	e33	432	432	Reference		
FHS_C2	Baseline	Aβ40	Plasma	e4	402	402	1.457	2.213	5.10E-01
FHS_C2	Baseline	Aβ40	Plasma	e33	1982	1982	Reference		
FHS_C3	Baseline	Aβ40	Plasma	e4	370	370	−6.513	3.022	3.12E-02
FHS_C3	Baseline	Aβ40	Plasma	e33	1916	1916	Reference		
ARIC	Baseline	Aβ40	Plasma	e4	230	230	−12.561	6.078	3.90E-02
ARIC	Baseline	Aβ40	Plasma	e33	1083	1083	Reference		
Meta	Baseline	Aβ40	Plasma	e4	1112	1112	−2.091	1.608	1.93E-01
Meta	Baseline	Aβ40	Plasma	e33	5683	5683	Reference		
ADNI-1	Longitudinal	Aβ40	Plasma	e4	36	123	−3.345	6.555	6.10E-01
ADNI-1	Longitudinal	Aβ40	Plasma	e33	277	942	Reference		
Meta	Longitudinal	Aβ40	Plasma	e4	36	123	−3.345	6.555	6.10E-01
Meta	Longitudinal	Aβ40	Plasma	e33	277	942	Reference		
ADNI-1	Baseline	Aβ42	CSF	e4	17	17	0.162	0.106	1.27E-01
ADNI-1	Baseline	Aβ42	CSF	e33	125	125	Reference		
ADNI-2/GO	Baseline	Aβ42	CSF	e4	20	20	0.130	0.086	1.31E-01
ADNI-2/GO	Baseline	Aβ42	CSF	e33	134	134	Reference		
Meta	Baseline	Aβ42	CSF	e4	37	37	0.143	0.067	3.18E-02
Meta	Baseline	Aβ42	CSF	e33	259	259	Reference		
ADNI-1	Longitudinal	Aβ42	CSF	e4	19	42	0.142	0.150	3.42E-01
ADNI-1	Longitudinal	Aβ42	CSF	e33	151	374	Reference		
ADNI-2/GO	Longitudinal	Aβ42	CSF	e4	20	38	0.084	0.144	5.61E-01
ADNI-2/GO	Longitudinal	Aβ42	CSF	e33	142	231	Reference		
Meta	Longitudinal	Aβ42	CSF	e4	39	80	0.112	0.104	2.80E-01
Meta	Longitudinal	Aβ42	CSF	e33	293	605	Reference		
ADNI-1	Baseline	Aβ42	Plasma	e4	36	36	0.278	2.062	8.93E-01
ADNI-1	Baseline	Aβ42	Plasma	e33	273	273	Reference		
FHS_C1	Baseline	Aβ42	Plasma	e4	74	74	−1.185	1.499	4.30E-01

FHS_C1	Baseline	Aβ42	Plasma	e33	432	432	Reference		
FHS_C2	Baseline	Aβ42	Plasma	e4	402	402	0.228	0.567	6.88E-01
FHS_C2	Baseline	Aβ42	Plasma	e33	1982	1982	Reference		
FHS_C3	Baseline	Aβ42	Plasma	e4	370	370	−0.268	0.565	6.36E-01
FHS_C3	Baseline	Aβ42	Plasma	e33	1916	1916	Reference		
ARIC	Baseline	Aβ42	Plasma	e4	228	228	−0.198	0.830	8.12E-01
ARIC	Baseline	Aβ42	Plasma	e33	1076	1076	Reference		
Meta	Baseline	Aβ42	Plasma	e4	1110	1110	−0.105	0.346	7.61E-01
Meta	Baseline	Aβ42	Plasma	e33	5679	5679	Reference		
ADNI-1	Longitudinal	Aβ42	Plasma	e4	36	123	−0.982	1.751	5.75E-01
ADNI-1	Longitudinal	Aβ42	Plasma	e33	277	950	Reference		
Meta	Longitudinal	Aβ42	Plasma	e4	36	123	−0.982	1.751	5.75E-01
Meta	Longitudinal	Aβ42	Plasma	e33	277	950	Reference		
ADNI-1	Baseline	Tau	CSF	e4	24	24	−0.003	0.093	9.71E-01
ADNI-1	Baseline	Tau	CSF	e33	149	149	Reference		
ADNI-2/GO	Baseline	Tau	CSF	e4	31	31	−0.111	0.071	1.17E-01
ADNI-2/GO	Baseline	Tau	CSF	e33	192	192	Reference		
Meta	Baseline	Tau	CSF	e4	55	55	−0.072	0.056	2.02E-01
Meta	Baseline	Tau	CSF	e33	341	341	Reference		
ADNI-1	Longitudinal	Tau	CSF	e4	25	59	0.013	0.120	9.11E-01
ADNI-1	Longitudinal	Tau	CSF	e33	165	433	Reference		
ADNI-2/GO	Longitudinal	Tau	CSF	e4	31	61	−0.106	0.101	2.93E-01
ADNI-2/GO	Longitudinal	Tau	CSF	e33	194	334	Reference		
Meta	Longitudinal	Tau	CSF	e4	56	120	−0.057	0.077	4.64E-01
Meta	Longitudinal	Tau	CSF	e33	359	767	Reference		
ADNI-1	Baseline	Tau	Plasma	e4	32	32	−0.127	0.111	2.52E-01
ADNI-1	Baseline	Tau	Plasma	e33	223	223	Reference		
FHS_C1	Baseline	Tau	Plasma	e4	21	21	−0.127	0.055	2.12E-02
FHS_C1	Baseline	Tau	Plasma	e33	93	93	Reference		
FHS_C2	Baseline	Tau	Plasma	e4	350	350	0.011	0.020	5.77E-01
FHS_C2	Baseline	Tau	Plasma	e33	1721	1721	Reference		
FHS_C3	Baseline	Tau	Plasma	e4	370	370	0.037	0.019	4.80E-02
FHS_C3	Baseline	Tau	Plasma	e33	1914	1914	Reference		
Meta	Baseline	Tau	Plasma	e4	773	773	0.014	0.013	2.88E-01
Meta	Baseline	Tau	Plasma	e33	3951	3951	Reference		

ARIC is the Atherosclerosis Risk in Communities Study. FHS_C1, FHS_C2, and FHS_C3 denote the Framingham Heart Study parental, offspring, and grandchildren cohorts, respectively. ADNI-1 and ADNI-2/GO denote the Alzheimer's disease Neuroimaging Initiative initial and extended cohorts, respectively. Meta shows the results of meta-analysis. Meta-analysis field was shown for consistency for each biomarker even if the analysis was performed in one study only. Column "Type" indicates biomarker measurements at baseline or longitudinally. Abbreviations: CSF: cerebrospinal fluid; SE: standard error. The APOE ε2 allele was defined as e22 and e23 genotypes. A gamma general linear model with a log link function was used for all biomarkers except Aβ40 and Aβ42 measured in plasma.

Supplementary Table 5. Associations of compound genotypes with Alzheimer's disease (AD) Aβ42 and tau biomarkers measured at baseline.

Study	Biomarker	Source	Genotype	*N*	Beta	SE	*P* value
ADNI-1	Aβ42	CSF	0XY	30	−0.029	0.079	7.14E-01
ADNI-1	Aβ42	CSF	100+200	31	−0.347	0.084	6.82E-05
ADNI-1	Aβ42	CSF	111+222	101	−0.411	0.055	1.64E-12
ADNI-1	Aβ42	CSF	1XY+2XY	138	−0.450	0.050	1.08E-16
ADNI-1	Aβ42	CSF	000	112	Reference		
ADNI-2/GO	Aβ42	CSF	0XY	29	0.078	0.077	3.13E-01
ADNI-2/GO	Aβ42	CSF	100+200	25	−0.208	0.078	8.82E-03
ADNI-2/GO	Aβ42	CSF	111+222	77	−0.268	0.056	3.06E-06
ADNI-2/GO	Aβ42	CSF	1XY+2XY	104	−0.288	0.053	1.77E-07
ADNI-2/GO	Aβ42	CSF	000	125	Reference		
Meta	Aβ42	CSF	0XY	59	0.026	0.055	6.37E-01
Meta	Aβ42	CSF	100+200	56	−0.272	0.057	2.13E-06
Meta	Aβ42	CSF	111+222	178	−0.341	0.039	2.46E-18
Meta	Aβ42	CSF	1XY+2XY	242	−0.373	0.037	2.18E-24
Meta	Aβ42	CSF	000	237	Reference		
ADNI-1	Aβ42	Plasma	0XY	48	−2.137	1.752	2.23E-01
ADNI-1	Aβ42	Plasma	100+200	51	−1.342	1.769	4.49E-01
ADNI-1	Aβ42	Plasma	111+222	197	−2.598	1.067	1.53E-02
ADNI-1	Aβ42	Plasma	1XY+2XY	252	−2.418	1.012	1.73E-02
ADNI-1	Aβ42	Plasma	000	261	Reference		
FHS_C1	Aβ42	Plasma	0XY	60	−2.829	1.588	7.54E-02
FHS_C1	Aβ42	Plasma	100+200	22	−4.084	2.621	1.20E-01
FHS_C1	Aβ42	Plasma	111+222	50	−4.227	1.776	1.78E-02
FHS_C1	Aβ42	Plasma	1XY+2XY	77	−3.850	1.442	7.86E-03
FHS_C1	Aβ42	Plasma	000	344	Reference		
FHS_C2	Aβ42	Plasma	0XY	354	−0.582	0.585	3.20E-01
FHS_C2	Aβ42	Plasma	100+200	133	−2.231	0.931	1.66E-02
FHS_C2	Aβ42	Plasma	111+222	397	−1.670	0.573	3.59E-03
FHS_C2	Aβ42	Plasma	1XY+2XY	504	−2.093	0.516	5.12E-05
FHS_C2	Aβ42	Plasma	000	1843	Reference		
FHS_C3	Aβ42	Plasma	0XY	292	1.460	0.626	1.98E-02
FHS_C3	Aβ42	Plasma	100+200	128	−1.586	0.908	8.07E-02
FHS_C3	Aβ42	Plasma	111+222	512	−0.848	0.494	8.63E-02
FHS_C3	Aβ42	Plasma	1XY+2XY	615	−0.618	0.462	1.81E-01
FHS_C3	Aβ42	Plasma	000	1994	Reference		
ARIC	Aβ42	Plasma	0XY	197	1.809	0.860	3.56E-02
ARIC	Aβ42	Plasma	100+200	62	−4.463	1.463	2.34E-03
ARIC	Aβ42	Plasma	111+222	306	−2.109	0.724	3.65E-03
ARIC	Aβ42	Plasma	1XY+2XY	373	−2.304	0.671	6.15E-04
ARIC	Aβ42	Plasma	000	1107	Reference		
Meta	Aβ42	Plasma	0XY	951	0.351	0.364	3.35E-01
Meta	Aβ42	Plasma	100+200	396	−2.305	0.551	2.82E-05
Meta	Aβ42	Plasma	111+222	1462	−1.582	0.312	4.14E-07
Meta	Aβ42	Plasma	1XY+2XY	1821	−1.656	0.287	7.98E-09
Meta	Aβ42	Plasma	000	5549	Reference		
ADNI-1	Tau	CSF	0XY	31	0.158	0.075	3.61E-02
ADNI-1	Tau	CSF	100+200	30	0.234	0.080	3.99E-03
ADNI-1	Tau	CSF	111+222	105	0.247	0.049	8.28E-07
ADNI-1	Tau	CSF	1XY+2XY	142	0.244	0.043	4.96E-08
ADNI-1	Tau	CSF	000	142	Reference		
ADNI-2/GO	Tau	CSF	0XY	39	0.031	0.068	6.46E-01
ADNI-2/GO	Tau	CSF	100+200	26	0.232	0.077	3.05E-03
ADNI-2/GO	Tau	CSF	111+222	84	0.342	0.050	5.86E-11

ADNI-2/GO	Tau	CSF	1XY+2XY	111	0.355	0.048	1.38E-12
ADNI-2/GO	Tau	CSF	000	184	Reference		
Meta	Tau	CSF	0XY	70	0.088	0.050	7.97E-02
Meta	Tau	CSF	100+200	56	0.233	0.056	2.85E-05
Meta	Tau	CSF	111+222	189	0.293	0.035	4.87E-17
Meta	Tau	CSF	1XY+2XY	253	0.294	0.032	6.75E-20
Meta	Tau	CSF	000	326	Reference		
ADNI-1	Tau	Plasma	0XY	39	−0.059	0.099	5.49E-01
ADNI-1	Tau	Plasma	100+200	38	0.048	0.102	6.37E-01
ADNI-1	Tau	Plasma	111+222	156	0.085	0.059	1.53E-01
ADNI-1	Tau	Plasma	1XY+2XY	204	0.117	0.059	4.83E-02
ADNI-1	Tau	Plasma	000	216	Reference		
FHS_C1	Tau	Plasma	0XY	17	−0.024	0.092	7.93E-01
FHS_C1	Tau	Plasma	100+200	5	−0.040	0.052	4.43E-01
FHS_C1	Tau	Plasma	111+222	9	−0.099	0.133	4.58E-01
FHS_C1	Tau	Plasma	1XY+2XY	14	−0.172	0.103	9.89E-02
FHS_C1	Tau	Plasma	000	91	Reference		
FHS_C2	Tau	Plasma	0XY	300	0.030	0.020	1.27E-01
FHS_C2	Tau	Plasma	100+200	118	−0.054	0.030	7.47E-02
FHS_C2	Tau	Plasma	111+222	357	−0.022	0.019	2.34E-01
FHS_C2	Tau	Plasma	1XY+2XY	448	−0.008	0.017	6.55E-01
FHS_C2	Tau	Plasma	000	1615	Reference		
FHS_C3	Tau	Plasma	0XY	291	−0.005	0.018	7.86E-01
FHS_C3	Tau	Plasma	100+200	128	−0.047	0.026	7.72E-02
FHS_C3	Tau	Plasma	111+222	511	−0.007	0.015	6.07E-01
FHS_C3	Tau	Plasma	1XY+2XY	614	−0.002	0.013	8.88E-01
FHS_C3	Tau	Plasma	000	1991	Reference		
Meta	Tau	Plasma	0XY	647	0.009	0.013	4.91E-01
Meta	Tau	Plasma	100+200	289	−0.045	0.018	1.28E-02
Meta	Tau	Plasma	111+222	1033	−0.010	0.011	3.66E-01
Meta	Tau	Plasma	1XY+2XY	1280	−0.002	0.010	8.42E-01
Meta	Tau	Plasma	000	3913	Reference		

ARIC is the Atherosclerosis Risk in Communities Study. FHS_C1, FHS_C2, and FHS_C3 denote the Framingham Heart Study parental, offspring, and grandchildren cohorts, respectively. ADNI-1 and ADNI-2/GO denote the Alzheimer's disease Neuroimaging Initiative initial and extended cohorts, respectively. Meta shows the results of meta-analysis. Meta-analysis field was shown for consistency for each biomarker even if the analysis was performed in one study only. Column "Genotype" shows compound genotypes encoded by triples of numbers and X and Y letters. Numbers show the counts of minor alleles (i.e., 0, 1, 2) in rs429358_T/c, rs2075650_A/g or rs12721046_G/a SNP, in that order. The upper/lower case denotes here major/minor allele. The most frequent 000 genotype denotes the major allele homozygote for all three SNPs, i.e., rs429358_TT, rs2075650_AA, rs12721046_GG. The 100+200 genotype indicates rs429358_Tc, rs2075650_AA, rs12721046_GG (100) and rs429358_cc, rs2075650_AA, rs12721046_GG (200). The 111+222 genotype denotes rs429358_Tc, rs2075650_Ag, rs12721046_Ga (111) and rs429358_cc, rs2075650_gg, rs12721046_aa (222). Letters X and Y indicate aggregation of minor alleles of rs2075650 and rs12721046, respectively. Genotype 0XY aggregates all non-e4 genotypes except 000. The 1XY+2XY genotype aggregates rs429358_Tc (1) and rs429358_cc (2) and all genotypes of rs2075650 (X) and rs12721046 (Y), except major allele homozygote of both SNPs, rs2075650_AA and rs12721046_GG (00), because it is included in the 100+200 genotype. Column "SE" shows standard error. A gamma general linear model with a log link function was used for all biomarkers except Aβ42 measured in plasma.

Supplementary Table 6. Comparative analysis of the associations of the selected compound genotypes with Alzheimer's disease (AD) Aβ42 and tau biomarkers.

Study	Biomarker	Source	Genotype	*N*	Beta	SE	*P* value
ADNI-1	Aβ42	CSF	111+222	101	−0.047	0.081	5.62E-01
ADNI-1	Aβ42	CSF	1XY+2XY	138	−0.087	0.077	2.64E-01
ADNI-1	Aβ42	CSF	100+200	31	Reference		
ADNI-2/GO	Aβ42	CSF	111+222	77	−0.065	0.095	4.99E-01
ADNI-2/GO	Aβ42	CSF	1XY+2XY	104	−0.097	0.096	3.14E-01
ADNI-2/GO	Aβ42	CSF	100+200	25	Reference		
Meta	Aβ42	CSF	111+222	178	−0.054	0.062	3.77E-01
Meta	Aβ42	CSF	1XY+2XY	242	−0.091	0.060	1.32E-01
Meta	Aβ42	CSF	100+200	56	Reference		
ADNI-1	Aβ42	Plasma	111+222	197	−1.200	1.626	4.61E-01
ADNI-1	Aβ42	Plasma	1XY+2XY	252	−1.189	1.647	4.71E-01
ADNI-1	Aβ42	Plasma	100+200	51	Reference		
FHS_C1	Aβ42	Plasma	111+222	50	0.011	2.532	9.96E-01
FHS_C1	Aβ42	Plasma	1XY+2XY	77	0.004	2.163	9.98E-01
FHS_C1	Aβ42	Plasma	100+200	22	Reference		
FHS_C2	Aβ42	Plasma	111+222	397	0.442	0.962	6.46E-01
FHS_C2	Aβ42	Plasma	1XY+2XY	504	0.051	0.914	9.56E-01
FHS_C2	Aβ42	Plasma	100+200	133	Reference		
FHS_C3	Aβ42	Plasma	111+222	512	0.706	0.997	4.79E-01
FHS_C3	Aβ42	Plasma	1XY+2XY	615	0.939	0.992	3.44E-01
FHS_C3	Aβ42	Plasma	100+200	128	Reference		
ARIC	Aβ42	Plasma	111+222	306	2.508	1.460	8.66E-02
ARIC	Aβ42	Plasma	1XY+2XY	373	2.302	1.455	1.14E-01
ARIC	Aβ42	Plasma	100+200	62	Reference		
Meta	Aβ42	Plasma	111+222	1462	0.619	0.569	2.77E-01
Meta	Aβ42	Plasma	1XY+2XY	1821	0.509	0.553	3.57E-01
Meta	Aβ42	Plasma	100+200	396	Reference		
ADNI-1	Tau	CSF	111+222	105	0.041	0.072	5.70E-01
ADNI-1	Tau	CSF	1XY+2XY	142	0.030	0.066	6.50E-01
ADNI-1	Tau	CSF	100+200	30	Reference		
ADNI-2/GO	Tau	CSF	111+222	84	0.107	0.092	2.50E-01
ADNI-2/GO	Tau	CSF	1XY+2XY	111	0.121	0.095	2.04E-01
ADNI-2/GO	Tau	CSF	100+200	26	Reference		
Meta	Tau	CSF	111+222	189	0.066	0.057	2.46E-01
Meta	Tau	CSF	1XY+2XY	253	0.060	0.054	2.70E-01
Meta	Tau	CSF	100+200	56	Reference		
ADNI-1	Tau	Plasma	111+222	156	0.030	0.085	7.26E-01
ADNI-1	Tau	Plasma	1XY+2XY	204	0.063	0.098	5.18E-01
ADNI-1	Tau	Plasma	100+200	38	Reference		
FHS_C1	Tau	Plasma	111+222	9	0.075	0.288	8.02E-01
FHS_C1	Tau	Plasma	1XY+2XY	14	0.000	0.342	1.00E+00
FHS_C1	Tau	Plasma	100+200	5	Reference		

FHS_C2	Tau	Plasma	111+222	357	0.027	0.035	4.33E-01
FHS_C2	Tau	Plasma	1XY+2XY	448	0.053	0.032	1.01E-01
FHS_C2	Tau	Plasma	100+200	118	Reference		
FHS_C3	Tau	Plasma	111+222	511	0.037	0.029	2.03E-01
FHS_C3	Tau	Plasma	1XY+2XY	614	0.041	0.028	1.39E-01
FHS_C3	Tau	Plasma	100+200	128	Reference		
Meta	Tau	Plasma	111+222	1033	0.033	0.021	1.25E-01
Meta	Tau	Plasma	1XY+2XY	1280	0.047	0.021	2.27E-02
Meta	Tau	Plasma	100+200	289	Reference		

ARIC is the Atherosclerosis Risk in Communities Study. FHS_C1, FHS_C2, and FHS_C3 denote the Framingham Heart Study parental, offspring, and grandchildren cohorts, respectively. ADNI-1 and ADNI-2/GO denote the Alzheimer's disease Neuroimaging Initiative initial and extended cohorts, respectively. Meta shows the results of meta-analysis. Meta-analysis field was shown for consistency for each biomarker even if the analysis was performed in one study only. Column "Genotype" shows compound genotypes encoded by triples of numbers and X and Y letters. Numbers show the counts of minor alleles (i.e., 0, 1, 2) in rs429358_T/c, rs2075650_A/g or rs12721046_G/a SNP, in that order. The upper/lower case denotes here major/minor allele. The most frequent 000 genotype denotes the major allele homozygote for all three SNPs, i.e., rs429358_TT, rs2075650_AA, rs12721046_GG. The 100+200 genotype indicates rs429358_Tc, rs2075650_AA, rs12721046_GG (100) and rs429358_cc, rs2075650_AA, rs12721046_GG (200). The 111+222 genotype denotes rs429358_Tc, rs2075650_Ag, rs12721046_Ga (111) and rs429358_cc, rs2075650_gg, rs12721046_aa (222). Letters X and Y indicate aggregation of minor alleles of rs2075650 and rs12721046, respectively. The 1XY+2XY genotype aggregates rs429358_Tc (1) and rs429358_cc (2) and all genotypes of rs2075650 (X) and rs12721046 (Y), except major allele homozygote of both SNPs, rs2075650_AA and rs12721046_GG (00), because it is included in the 100+200 genotype. Column "SE" shows standard error. A gamma general linear model with a log link function was used for all biomarkers except Aβ42 measured in plasma.

Supplementary Table 7. Comparative analysis of the associations of the selected compound genotypes with Alzheimer's disease (AD) Aβ42 and tau biomarkers with carriers of the ε2 allele excluded.

Study	Biomarker	Source	Genotype	*N*	Beta	SE	*P* value
ADNI-1	Aβ42	CSF	111+222	97	−0.060	0.082	4.67E-01
ADNI-1	Aβ42	CSF	1XY+2XY	134	−0.099	0.078	2.06E-01
ADNI-1	Aβ42	CSF	100+200	31	Reference		
ADNI-2/GO	Aβ42	CSF	111+222	75	−0.054	0.094	5.67E-01
ADNI-2/GO	Aβ42	CSF	1XY+2XY	102	−0.091	0.096	3.46E-01
ADNI-2/GO	Aβ42	CSF	100+200	25	Reference		
Meta	Aβ42	CSF	111+222	172	−0.057	0.062	3.54E-01
Meta	Aβ42	CSF	1XY+2XY	236	−0.096	0.061	1.14E-01
Meta	Aβ42	CSF	100+200	56	Reference		
ADNI-1	Aβ42	Plasma	111+222	187	−1.291	1.661	4.38E-01
ADNI-1	Aβ42	Plasma	1XY+2XY	241	−1.261	1.685	4.55E-01
ADNI-1	Aβ42	Plasma	100+200	50	Reference		
FHS_C1	Aβ42	Plasma	111+222	43	0.658	2.716	8.09E-01
FHS_C1	Aβ42	Plasma	1XY+2XY	70	0.840	2.261	7.11E-01
FHS_C1	Aβ42	Plasma	100+200	21	Reference		
FHS_C2	Aβ42	Plasma	111+222	362	0.334	1.035	7.47E-01
FHS_C2	Aβ42	Plasma	1XY+2XY	465	−0.067	0.981	9.46E-01
FHS_C2	Aβ42	Plasma	100+200	116	Reference		
FHS_C3	Aβ42	Plasma	111+222	460	0.870	1.068	4.16E-01
FHS_C3	Aβ42	Plasma	1XY+2XY	561	1.088	1.063	3.06E-01
FHS_C3	Aβ42	Plasma	100+200	113	Reference		
ARIC	Aβ42	Plasma	111+222	273	2.587	1.474	8.02E-02
ARIC	Aβ42	Plasma	1XY+2XY	338	2.458	1.476	9.66E-02

ARIC	Aβ42	Plasma	100+200	59	Reference		
Meta	Aβ42	Plasma	111+222	1325	0.682	0.601	2.57E-01
Meta	Aβ42	Plasma	1XY+2XY	1675	0.594	0.584	3.09E-01
Meta	Aβ42	Plasma	100+200	359	Reference		
ADNI-1	Tau	CSF	111+222	100	0.036	0.071	6.16E-01
ADNI-1	Tau	CSF	1XY+2XY	137	0.023	0.065	7.22E-01
ADNI-1	Tau	CSF	100+200	30	Reference		
ADNI-2/GO	Tau	CSF	111+222	82	0.116	0.092	2.11E-01
ADNI-2/GO	Tau	CSF	1XY+2XY	109	0.128	0.094	1.79E-01
ADNI-2/GO	Tau	CSF	100+200	26	Reference		
Meta	Tau	CSF	111+222	182	0.066	0.056	2.43E-01
Meta	Tau	CSF	1XY+2XY	246	0.057	0.054	2.89E-01
Meta	Tau	CSF	100+200	56	Reference		
ADNI-1	Tau	Plasma	111+222	150	0.033	0.085	6.99E-01
ADNI-1	Tau	Plasma	1XY+2XY	197	0.067	0.099	4.97E-01
ADNI-1	Tau	Plasma	100+200	38	Reference		
FHS_C1	Tau	Plasma	111+222	8	0.079	0.299	7.98E-01
FHS_C1	Tau	Plasma	1XY+2XY	13	0.001	0.259	9.97E-01
FHS_C1	Tau	Plasma	100+200	5	Reference		
FHS_C2	Tau	Plasma	111+222	329	0.022	0.037	5.49E-01
FHS_C2	Tau	Plasma	1XY+2XY	417	0.050	0.034	1.46E-01
FHS_C2	Tau	Plasma	100+200	104	Reference		
FHS_C3	Tau	Plasma	111+222	459	0.031	0.029	2.93E-01
FHS_C3	Tau	Plasma	1XY+2XY	560	0.038	0.028	1.84E-01
FHS_C3	Tau	Plasma	100+200	113	Reference		
Meta	Tau	Plasma	111+222	946	0.028	0.022	2.03E-01
Meta	Tau	Plasma	1XY+2XY	1187	0.044	0.021	4.06E-02
Meta	Tau	Plasma	100+200	260	Reference		

ARIC is the Atherosclerosis Risk in Communities Study. FHS_C1, FHS_C2, and FHS_C3 denote the Framingham Heart Study parental, offspring, and grandchildren cohorts, respectively. ADNI-1 and ADNI-2/GO denote the Alzheimer's disease Neuroimaging Initiative initial and extended cohorts, respectively. Meta shows the results of meta-analysis. Meta-analysis field was shown for consistency for each biomarker even if the analysis was performed in one study only. Column "Genotype" shows compound genotypes encoded by triples of numbers and X and Y letters. Numbers show the counts of minor alleles (i.e., 0, 1, 2) in rs429358_T/c, rs2075650_A/g or rs12721046_G/a SNP, in that order. The upper/lower case denotes here major/minor allele. The most frequent 000 genotype denotes the major allele homozygote for all three SNPs, i.e., rs429358_TT, rs2075650_AA, rs12721046_GG. The 100+200 genotype indicates rs429358_Tc, rs2075650_AA, rs12721046_GG (100) and rs429358_cc, rs2075650_AA, rs12721046_GG (200). The 111+222 genotype denotes rs429358_Tc, rs2075650_Ag, rs12721046_Ga (111) and rs429358_cc, rs2075650_gg, rs12721046_aa (222). Letters X and Y indicate aggregation of minor alleles of rs2075650 and rs12721046, respectively. The 1XY+2XY genotype aggregates rs429358_Tc (1) and rs429358_cc (2) and all genotypes of rs2075650 (X) and rs12721046 (Y), except major allele homozygote of both SNPs, rs2075650_AA and rs12721046_GG (00), because it is included in the 100+200 genotype. Column "SE" shows standard error. A gamma general linear model with a log link function was used for all biomarkers except Aβ42 measured in plasma.
